# SARS-CoV-2 Nucleocapsid Protein Mutations Found in Switzerland Disrupt N-Gene Amplification in Commonly Used Multiplex RT-PCR Assay

**DOI:** 10.3390/pathogens12121383

**Published:** 2023-11-24

**Authors:** Dominique Hilti, Faina Wehrli, Anna Roditscheff, Martin Risch, Lorenz Risch, Adrian Egli, Thomas Bodmer, Nadia Wohlwend

**Affiliations:** 1 Laboratory Dr. Risch, 9470 Buchs, Switzerland; faina.wehrli@risch.ch (F.W.); martin.risch@risch.ch (M.R.); lorenz.risch@risch.ch (L.R.); nadia.wohlwend@risch.ch (N.W.); 2 Faculty of Medical Science, Private University in the Principality of Liechtenstein (UFL), 9495 Triesen, Liechtenstein; anna.roditscheff@risch.ch; 3 Laboratory Dr. Risch, Liebefeld, 3097 Köniz, Switzerland; 4 Zentrallabor, Kantonsspital Graubünden, 7000 Chur, Switzerland; 5 Department of Medical Microbiology, University of Zurich, 8006 Zurich, Switzerland; aegli@imm.uzh.ch

**Keywords:** SARS-CoV-2, target failure, polymerase chain reaction, whole-genome sequencing

## Abstract

At the end of 2021, we observed an increase in N-gene target failures (NGTF) with the TaqPathTM COVID-19 CE-IVD RT-PCR Kit from Thermo Fisher Scientific (TaqPath). We subsequently used whole-genome sequencing (Oxford Nanopore Technology) to identify potential issues with N-gene PCR efficacy. Among 168,101 positive samples with a cycle threshold (CT) value <30 from August 2021 to May 2022, 194 specimens without N-gene amplification by PCR were identified (0.12%). Most NGTF samples originated from a wave of infection attributable to the Delta variant (B.1.617.2) and its sublineages. Sequencing revealed the nucleotide substitution G28922T (A217S) in 151 samples (88.8%). The substitution G215C, a hallmark mutation for Delta lineages, was concurrently present in all of these samples. Ten samples (5.9%) carried the deletion 28,913–28,918 (del214/215), eight samples (4.7%) the deletion 28,913–28,915 (del214) and one sample (0.6%) the deletion 28,892–28,930 (del207–219). Samples showing intact N-gene amplification by PCR lacked these specific mutations, but delayed-type amplification (i.e., partial or pNGTF) was attributable to the exclusive presence of A217S. As the N gene is a common target in many RT-PCR methods for SARS-CoV-2, an in-depth analysis of single-target failures using a combination with viral whole genome sequencing may allow for the identification of diagnostic flaws and eventual new variants.

## 1. Introduction

Severe acute respiratory syndrome coronavirus 2 (SARS-CoV-2), first detected in Wuhan, China, in winter 2019, is the causative agent of the COVID-19 pandemic and is responsible for more than 768 million confirmed infections and 6.9 million deaths worldwide as of July 2023 [1]. The first isolation of the novel agent on 7 January 2020, and sharing of its genetic sequence with the public by the Chinese authorities [2] led to rapid development of numerous specific PCR-based diagnostic assays.

Phylogenetic estimates for the nucleotide evolutionary rate of SARS-CoV-2 range from 7 × 10^−4^ to 1.1 × 10^−3^ substitutions (subs) per site per year [3,4]. With current data, the Nextstrain website estimates the annual nucleotide evolution rate at 1.07 × 10^−3^ subs/site/year, which is an increase from the 8 × 10^−4^ subs/site/year reported in July 2021 [5]. A virus’s genome becomes increasingly mutated the more it spreads over a population, increasing the possibility of new, highly contagious variants emerging that might spread around the world. Such variants of concern (VoCs), as classified by the World Health Organization, have been causing multiple waves of COVID-19 worldwide. To date, many mutations in the SARS-CoV-2 genome that have the potential to increase viral transmission, increase the severity of disease, reduce the effectiveness of treatments or vaccines and/or interfere with diagnostic detection have accumulated [4,5].

As PCR-based diagnostic assays have been at the forefront of identifying cases of COVID-19, interference from genetic mutations might lead to diagnostic escape, emphasizing the need to test for multiple targets. As in the case of missing SARS-CoV-2 target signals in PCR, diagnostic interference may indicate the presence of new variants, as has been described for the Alpha variant (B.1.1.7) in 2020 [6]. In this case, the viral genome harbors the amino acid deletion H69/V70, which leads to amplification failure of the S-gene target with TaqPath^TM^ COVID-19 CE-IVD RT-PCR Kit (Thermo Fisher, Lucerne, Switzerland). This S-gene target failure (SGTF), and its absence, was subsequently used as a surrogate marker to estimate the frequency and transmissibility of dominant variants, as it is a hallmark mutation of Alpha and Omicron BA.1, BA.3, BA.4 and BA.5 variants [7,8].

A variety of different mutations that cause target failure in different diagnostic assays have been described [9] and are summarized in Table A1 (Appendix A). The G214 and G215 deletions were first observed in a single sample from a hospitalized Italian man that showed N-gene target failure with the Allplex^TM^ SARS-CoV-2 Assay [10]. The C29200T nucleotide substitution, which inhibits N-gene target detection in GeneXpert Xpress, was first described in a sample from a female patient in Germany after her return from Romania [11].

Here, we report another novel mutation that interferes with the N-gene target in a commonly used multiplex RT-PCR assay.

## 2. Materials and Methods

### 2.1. Patients and Samples

Dr. Risch Laboratories provides SARS-CoV-2 PCR testing for all 7 major regions of Switzerland and the Principality of Liechtenstein. Testing was performed for symptomatic individuals as well as asymptomatic carriers in the form of mass testing programs. The majority of specimens consisted of nasopharyngeal swabs and saliva samples. Other specimens, such as sputum and bronchial secretions, were rarely tested.

A multitude of different diagnostic PCR assays for SARS-CoV-2 were used in our laboratory (e.g., Cepheid’s Xpert^®^ Xpress SARS-CoV-2, VIASURE SARS-CoV-2 (N1 + N2) Real Time PCR Detection Kit for BD MAX or TaqPath COVID-19 CE-IVD RT-PCR Kit by Thermo Fisher, Lunau, Switzerland (TaqPath)); however, only samples tested with the TaqPath Kit were included in this study and further investigated. Testing was performed according to the manufacturer’s instructions using either the Quantstudio^TM^ 5 or Quantstudio^TM^ 7 qPCR System (Amplitude Solution). Complete N-gene target failure (NGTF) was defined as successful detection of both ORF1ab- and S-gene amplicons in the absence of amplification of the N-gene target. Additionally, the N-gene shift, i.e., the cycle threshold (CT) value difference between the N and ORF1ab genes, was calculated for triple-positive samples. Samples with an N-gene shift above 3 CT values were designated as partial N-gene target failure (pNGTF), taking into consideration cycle threshold variability of different targets within a single test performance [12].

(1) $$S-gene\;shift=CT\;\left(N\right)-CT\;\left(ORF1ab\right)$$

**Figure A1:**
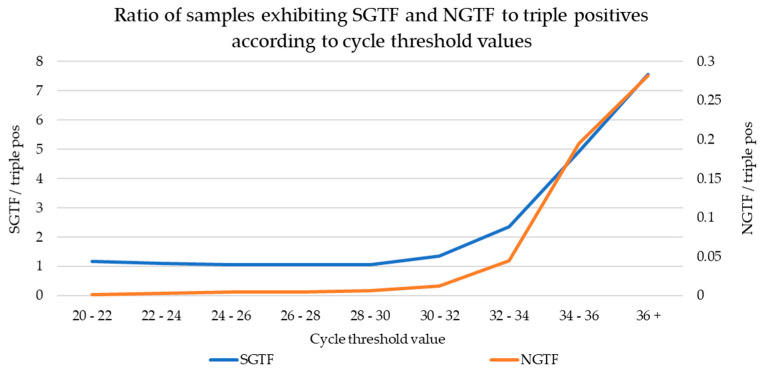
The ratio of samples presenting with S-gene target failure (SGTF) or N-gene target failure (NGTF) in relation to triple-positive samples. An increasing number of samples exhibiting SGTF or NGTF is observed when cycle threshold (CT) values for the ORF1ab target gene are greater than 30, as illustrated by the rising slope.

After initial observation of the NGTF phenomenon, samples were retrospectively analyzed from August 2021 to May 2022. Only samples with CT values less than 30 in the ORF1ab gene assay were included. Primarily, this decision was made because samples with lower viral load/higher CT values may show nonspecific target failure for any of the three target genes (Figure A1, Appendix A) and because whole-genome sequencing is rarely achievable with adequate coverage for samples with a CT value greater than 30. Finally, whole-genome sequencing was performed for all eligible samples using next-generation sequencing (NGS) by Oxford Nanopore Technology (ONT, Oxford, UK) using either the ARTIC Network V3 or ONT’s own Midnight protocol [13] according to the manufacturer’s instructions.

### 2.2. Data Collection and Analysis

According to article 2 of the Swiss Federal Act on Research involving Human Beings, an analysis on anonymized biological material and anonymized health data does not qualify as research in a strict sense of the law and approval of a cantonal ethics commission as well as informed consent can thus be waived. Only sampling date and time as well as sampling place (canton) of the sampled materials without any personal information of patients was used for this analysis, where descriptive statistics have been done with Microsoft Excel (Microsoft, Seattle, WA, USA) and Medcalc (Mariakerke, Belgium) for computations.

Analysis using the GridIon nanopore sequencer was performed with the wf-artic Nextflow workflow provided by Oxford Nanopore Technologies. Lineage assignment was based on the Phylogenetic Assignment of Named Global Outbreak Lineages (PANGOLIN) algorithm (version 4.0.6, pangolin-data v1.8) [14]. PANGOLIN is a computational tool to assign the most likely lineage to a given SARS-CoV-2 genome based on the PANGO nomenclature [15]. The PANGO nomenclature is a fine-scaled nomenclature system for SARS-CoV-2 that aims to identify epidemiologically relevant clusters at the leading edge of pandemic transmission.

All sequencing data were shared via the Swiss Pathogen Surveillance Platform [16]. The genome sequences in this study can be accessed at GISAID, Global Initiative on Sharing All Influenza Data [17], via gisaid.org/EPI_SET_230901zc (accessed on 1 September 2023).

### 2.3. GISAID

For mutational frequency calculations, the complete library of SARS-CoV-2 sequences at GISAID was accessed on the 19th of May 2022 through the GISAID homepage. Filtering was performed with the built-in filter functions for variants as well as amino acid (AA) substitutions. Selected VOCs were Alpha, Beta, Gamma, Delta and Omicron, and the search terms for AA substitutions were N_G215C and N_A217S, alone or in combination.

## 3. Results

### 3.1. Characteristics

From August 2021 to May 2022, a total of 1,334,687 specimens were tested for SARS-CoV-2. With 218,815 positive specimens, a cumulative positivity rate of 16.4% [95% CI, 16.3%, 16.5%] was calculated. A total of 168,101 samples were considered eligible (CT < 30, TaqPath), of which 0.12% (n = 194) showed complete absence of N-gene target amplification, i.e., NGTF. The frequency of NGTF varied between 0% and 1% per week during the observation period.

**Figure 1:**
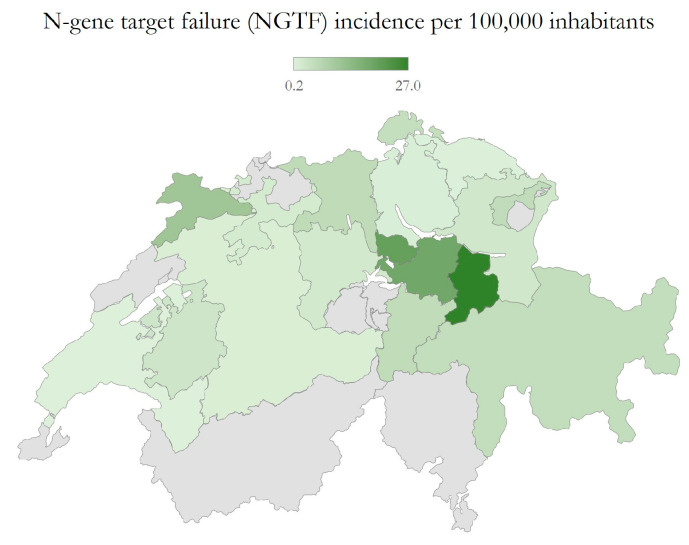
Distribution of samples presenting with complete N-gene target failure (NGTF) in TaqPath PCR across Switzerland expressed as incidence per 100.000 inhabitants. The highest numbers were found in the cantons of Aargau (n = 39), Schwyz (n = 30) and Zug (n = 26).

Samples with NGTF were found in 17 of 26 cantons of Switzerland, with the highest frequency in the cantons Aargau (n = 39), Schwyz (n = 30) and Zug (n = 26). Samples from cantons where no NGTF was observed accounted for less than 5% of the samples tested in our lab. The distribution of NGTF in Switzerland is shown in Figure 1.

**Figure 2:**
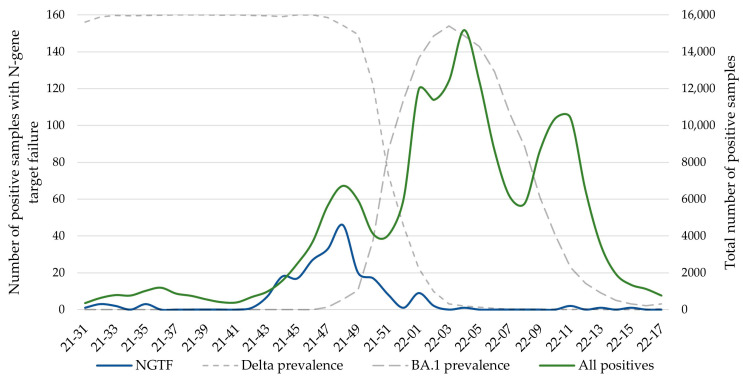
Comparison of the total number of positive samples and the number of positive samples with N-gene target failure. Although the occurrence of samples with NGTF correlated with emergence of the Delta variant, its frequency decreased with that of Omicron BA.1. Prevalence data for Switzerland (dashed lines) were obtained from GISAID and span from 0 to 100%.

The frequency of NGTF was found to coincide with the appearance of the Delta variant (B.1.617.2. *), whereas emergence of the two Omicron variants BA.1 and BA.2 (B.1.529. *) reduced its occurrence (Figure 2). The highest frequency of NGTF, at 1%, was observed in November 2021. A clear decrease then occurred during the introduction of the Omicron variant BA.1 in December 2021. Since then, NGTF has been observed only sporadically.

### 3.2. Genomic Analysis

The findings from whole-genome sequencing of samples with NGTF are summarized in Table 1. The most frequent mutation associated with NGTF with the TaqPath Kit was the amino acid substitution A217S (G28922T), at 88.8% (n = 151/170); the most frequent lineage was the Delta sub lineage AY.4 (B.1.617.2.4), at 68.5% (n = 102/149). Based on GISAID, the frequency of the AY.4 sublineage among all Delta lineages in Switzerland was 10.7% as of May 2022. A total of 97% of specimens presenting with NGTF and belonging to the AY.4 sublineage showed the A217S substitution. A PANGO lineage could be established for 149 sequences, but WGS was not performed sufficiently for 35 samples to be processed by PANGOLIN. Mutations in the N-gene region were registered nonetheless if coverage was adequate (n = 21). For 14 samples, no relevant genomic data from sequencing could be obtained, which is in line with previously reported success rates for NGS of SARS-CoV-2 genomes [18,19]. Ten samples could not be retrieved from the archive.

The sample assigned to the Omicron lineage BA.2 did not show the A217S mutation but rather a 13-fold deletion spanning N amino acids (AA) 207 to 219. This deletion was not found in any of the sequences belonging to Delta lineages, and as of July 2023, there were only 27 sequences at GISAID harboring all 13 deletions worldwide.

Frequency calculations for the A217S mutation appearance in different VOCs among sequences at GISAID showed that it was 5-fold more frequently observed in the Delta variant (0.08%) than in other variants (0.016%), as indicated in Table 2. Additionally, the combination of G215C and A217S was almost exclusively limited to the Delta lineage (Table 3).

### 3.3. Target Failure as a Result of Concurrent Mutation Presence

Our hypothesis was that the A217S mutation was responsible for complete NGTF with the TaqPath Kit. However, one sample assigned to the Omicron BA.2 lineage that was randomly sequenced for SARS-CoV-2 surveillance was found to have acquired the A217S mutation but did not present the NGTF phenomenon by PCR analysis. No artifacts or noise amplification that could explain a false-positive signal for N-gene amplification were observed in the qPCR plot. Sequencing of samples presenting with NGTF showed that all contained G215C in addition to A217S.

G215C is a hallmark mutation for all Delta sublineages but is not commonly observed within Omicron lineages. As in this case, the BA.2 sequence did not harbor this additional mutation. Nevertheless, this sample did display an N-gene shift of 3.9 CT values. On further investigation, sequences of six more samples were found in which A217S was present without G215C in routinely sequenced samples. All of them belong to Omicron variants BA.2 and BA.5. Their corresponding CT values revealed that all of them exhibited pNGTF, with a mean N-gene shift of 4.16 [3.42 to 4.89]. None of these sequences showed complete NGTF.

## 4. Discussion

We report that the nucleotide substitution G28922T (A217S) and deletions in the regions 28,913–28,918 (G214-, G215-) and 28,892–28,930 (del207–219) of SARS-CoV-2 are associated with NGTF with TaqPath Kit. Our findings suggest that the A217S substitution leads to NGTF only in the presence of G215C, a hallmark mutation for Delta lineages.

Three different amino acids and a deletion (glycine, cysteine, serine, -) have become established at position 215 of the nucleocapsid protein since the beginning of the pandemic [5]. The Delta lineage, however, is the only lineage incorporating cysteine at this position. As shown in this study, the frequency of NGTF decreased after disappearance of the Delta variant in Switzerland, and CT values of routinely sequenced samples with A217S but without G215C all showed pNGTF. We therefore propose that the sole presence of A217S leads to partial NGTF with delayed-type CT values and that only the combination of G215C and A217S, as it is present in the Delta variant, is associated with complete target failure of the N gene in the context of the TaqPath Kit. This may also help to explain the immediate reduction and continuous absence of NGTF among SARS-CoV-2-positive samples after the disappearance of the Delta variant.

Although the G214 and G215 deletions have already been reported to cause NGTF with the TaqPath Kit [20], our most frequent finding, the A217S mutation, has not been described until now. There are 5194 sequences containing only the amino acid substitution A217S and 3385 sequences containing both A217S and G215C that have been uploaded to GISAID as of July 2022. Approximately 75% of these were from Europe or North America. With a total of 11,264,134 sequences, the mutation occurs with a frequency of 0.045%.

S-gene target failure (SGTF), the first example of PCR target failure in the SARS-CoV-2 era, became prominent in the winter of 2020/2021 due to the dominance of the Alpha variant at that time. However, the spike deletion H69-/V70- had already appeared in several lineages, including the B.1.258 lineage [21,22]. Comparably, all GISAID sequences assigned to lineage B.1.618 either display the A217S substitution or exhibit coverage issues in this region, indicating that the substitution has already occurred in this lineage before. B.1.618, B.1.617, B.1.617.1 (Kappa) and B.1.36.29 emerged in India in the late 2020s, similar to the Delta variant (B.1.617.2), and were subsequently named the Delta variant family [23]. Based on this spatiotemporal information, it is possible to hypothesize that the A217S mutation was introduced to the Delta lineage by an early recombination event with the B.1.618 lineage.

The N gene is a common target in RT-PCR for SARS-CoV-2, and several mutations that interfere with N-gene detection in these assays have been described. In-depth analysis of single-target failures using a combination with viral whole-genome sequencing may allow for the identification of diagnostic flaws and eventual new variants.

## 5. Conclusions

In conclusion, we report several mutations in the SARS-CoV-2 nucleocapsid protein associated with N-gene target amplification failure with the TaqPath Kit. Although the G214 and G215 deletions and concurrent presence of A217S and G215C appear to be causative for complete NGTF, the exclusive presence of A217S appears to be associated with delayed-type N-gene CT values (pNGTF).

## Figures and Tables

**Table 1:** Summary of findings from whole-genome sequencing combined with lineage-specific population characteristics.

PANGO Lineage	#	Frequency (%)	CT (median)	G215C + A217S	G214-	G214/215-	Del207–219
AY.43	8	5.4	24.8	8			
AY.33	2	1.3	21.8			2	
AY.4	102	68.5	25.7	99		3	
B.1.617.2	3	2.0	26.0	2		1	
AY.4.3	4	2.7	23.9	4			
AY.46.6	1	0.7	21.8			1	
AY.125	11	7.4	23.4	11			
AY.98.1	5	3.4	25.4		5		
AY.36	9	6.0	25.4	9			
AY.122	1	0.7	25.6		1		
AY.43.4	2	1.3	22.5			2	
BA.2	1	0.7	21.8				1
na ^1^	21		28.8	18	2	1	
Total	170	100.0%		151	8	10	1

^1^ Abbreviations: na, not applicable.

**Table 2:** Calculated frequency of N amino acid substitution A217S among different variants of concern (VOCs) according to data from GISAID. Although the frequency among other variants was stable at a low level, it was found to increase 5-fold within the Delta lineage.

GISAID VOC	N_A217S	Total	Frequency
Alpha	193	1,188,205	0.016%
Beta	7	43,474	0.016%
Gamma	22	126,630	0.017%
Delta	3518	4,459,248	0.079%
Omicron	698	4,588,964	0.015%

**Table 3:** Total hits at GISAID per variant when filtered for N-gene mutations “A217S” or “G215C and A217S”.

GISAID VOC	N_A217S	N_G215C and N_A217S
Alpha	214	0
Beta	7	0
Gamma	22	0
Delta	3651	3515
Omicron	1358	3
Total	6020	3518

**Figure A2:**
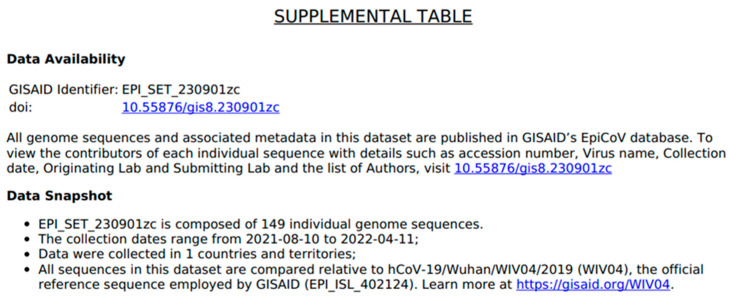
Data Availability Statement.

## Data Availability

The data presented in this study are openly available in GISAID’s EpiCoV database at doi: 10.557/gis8.230901zc.

## Appendix A

**Table A1:** Summary of previously published mutations affecting the N-gene efficacy in frequently employed SARS-CoV-2 PCR assays.

Mutation	Amino Acid	Effect on Assay	Assay	References
G29179T	synonymous	partial NGTF	Cepheid Xpert Xpress SARS-CoV-2	[24,25,26,27]
C29197T	synonymous	NGTF	Cepheid Xpert Xpress SARS-CoV-2	[28,29,30,31]
C29200T	synonymous	NGTF	Cepheid Xpert Xpress SARS-CoV-2	[11,28,29,30]
28,913–28,915	G214del	NGTF	TaqPath COVID-19 Combo Kit	[20]
28,916–28,918	G215del	NGTF	TaqPath COVID-19 Combo Kit	[20]
28,913–28,918	G214–G215 deletion	NGTF	Allplex^TM^ SARS-CoV-2 Assay, Seegene	[10]
